# Role of Arginase 1 from Myeloid Cells in Th2-Dominated Lung Inflammation

**DOI:** 10.1371/journal.pone.0061961

**Published:** 2013-04-24

**Authors:** Luke Barron, Amber M. Smith, Karim C. El Kasmi, Joseph E. Qualls, Xiaozhu Huang, Allen Cheever, Lee A. Borthwick, Mark S. Wilson, Peter J. Murray, Thomas A. Wynn

**Affiliations:** 1 Program in Barrier Immunity and Repair, Laboratory of Parasitic Diseases, National Institute of Allergy and Infectious Diseases, National Institutes of Health, Bethesda, Maryland, United States of America; 2 Departments of Infectious Diseases and Immunology, St. Jude Children’s Research Hospital, Memphis, Tennessee, United States of America; 3 Lung Biology Center, Department of Medicine, University of California San Francisco, San Francisco, California, United States of America; 4 Biomedical Research Institute, Rockville, Maryland, United States of America; 5 Tissue Fibrosis and Repair Group, Institute of Cellular Medicine, Medical School, Newcastle University, Newcastle upon Tyne, United Kingdom; 6 Division of Molecular Immunology, National Institute for Medical Research, MRC, London, United Kingdom; Louisiana State University Health Sciences Center, United States of America

## Abstract

Th2-driven lung inflammation increases Arginase 1 (Arg1) expression in alternatively-activated macrophages (AAMs). AAMs modulate T cell and wound healing responses and Arg1 might contribute to asthma pathogenesis by inhibiting nitric oxide production, regulating fibrosis, modulating arginine metabolism and restricting T cell proliferation. We used mice lacking Arg1 in myeloid cells to investigate the contribution of Arg1 to lung inflammation and pathophysiology. In six model systems encompassing acute and chronic Th2-mediated lung inflammation we observed neither a pathogenic nor protective role for myeloid-expressed Arg1. The number and composition of inflammatory cells in the airways and lungs, mucus secretion, collagen deposition, airway hyper-responsiveness, and T cell cytokine production were not altered if AAMs were deficient in Arg1 or simultaneously in both Arg1 and NOS2. Our results argue that Arg1 is a general feature of alternative activation but only selectively regulates Th2 responses. Therefore, attempts to experimentally or therapeutically inhibit arginase activity in the lung should be examined with caution.

## Introduction

Host defense against many helminth parasites requires Th2 immunity to kill, expel, or contain pathogens and to repair the injuries caused by infection. However, inappropriately controlled Th2 immune responses cause pathology such as the airway hyperresponsiveness, mucus secretion, inflammation, and lung remodeling and fibrosis that characterize asthma [1], [2]. The Th2 cytokines IL-4 and IL-13, which play a critical role in asthma, “alternatively” activate macrophages and induce receptors, cytokines, enzymes, and change phagocytosis, proliferation, and other cellular processes that enable AAMs to regulate their surrounding leukocytes, parenchymal cells, and environment [3]–[5]. Studies testing whether AAMs play a helpful, harmful, or unimportant role in asthma or other immune-mediated lung diseases reached contradictory conclusions [6]. Differences in experimental methods, particularly the choice of antigen and treatment regimen, likely accounts for some contradictory data, and negative feedback mechanisms may reconcile apparent differences if increased gene expression correlates with disease but inhibits pathology. Nevertheless, immune-modulating asthma therapies now undergoing clinical trial, such as those targeting IL-4, IL-13, chemokines, antibody receptors, PPAR-γ, or Toll-Like Receptors, are expected to alter the functions of macrophages even though it remains unclear what role macrophages play in the pathogenesis of asthma [7].

In this study we analyzed the role of Arginase 1 (Arg1) expressed by AAMs in six models of Th2-dominant lung inflammation. We focused on Arg1 because it is induced in asthma patients and experimental mouse models, can contribute to or suppress Th2-mediated pathology by different mechanisms, and represents a therapeutic target because its enzymatic mechanism is known in great detail, and existing drugs are known inhibitors of arginases in vivo [8], [9]. Arg1 is one of two enzymes that hydrolyze arginine to urea and ornithine, and is expressed constitutively by hepatocytes to play an essential role in the urea cycle [10]. Myeloid lineage cells also express Arg1 but, unlike the constitutive expression in the liver, myeloid Arg1 is predominantly regulated by exogenous stimuli [10], [11]. A second isoform, mitochondrial Arg2, is present in many cell types and can also be induced [9]. Compared to Arg1, however, Arg2 expression correlates weakly with lung inflammation, contributes little to the tissue arginase activity, and has not been identified as an inducible characteristic of AAMs [12]–[14]. Mouse and human arginase expression are not exactly matched: in cells isolated from human blood Arg1 has only been found in neutrophils [15], [16] although, importantly, human tissue macrophages have yet to be rigorously tested for Arg1 or Arg2 expression.

Two major cytokine pathways control macrophage-specific Arg1 expression. First, IL-4 and IL-13 induce Arg1 in macrophages as part of the IL-4Rα- and STAT6-dependent alternative activation program. Arg1+ AAMs abound in Th2-dominated diseases such as asthma, atopic dermatitis, and helminth and other parasite infections [3]. Second, tuberculosis and other intracellular bacterial infections trigger Arg1 expression in response to IL-6, G-CSF and IL-10 stimulation following Toll-like receptor activation [17], [18]. This second pathway requires STAT3 signaling in response to autocrine/paracrine cytokines but is independent of the IL-4Rα or STAT6 [18].

Since Th2 responses predominate in asthma and lung and airway inflammation the IL-4/13-IL-4Rα-Stat6 pathway is anticipated to drive Arg1 expression in these diseases, and an increase in Arg1 is associated with many types of lung inflammation [12], [14], [19]. These results are intriguing because, if arginases play a pathologic role in asthma, then arginase inhibitors might exhibit protective activity against Th2-driven disease [9], [20], [21]. Previous studies have accordingly examined arginine metabolism and tested pharmacological and RNAi-mediated inhibition of Arg1 in the lung [9], [14], [22]–[26] but did not specifically address the contribution of induced Arg1 expression in myeloid cells to the pathogenesis of Th2-driven lung inflammation. Set against these findings, lung pathology was unaffected by deleting Arg1 in the bone marrow-derived cells of chimeric mice, or in mice engineered to delete IL-4Ra in Lysozyme M-expressing cells so that IL-4 and IL-13 could not alternatively active macrophages and induce Arg1 [13], [27].

Here we sought to define essential functions of myeloid Arg1 in lung and airway inflammation driven by Th2-driven inflammation. We used genetically engineered mice lacking Arg1 in all hematopoietic and endothelial cells backcrossed to a C57BL/6 or BALB/c background [17]. These mice express a transgenic Cre recombinase under the control of the Tie2 promoter and *Arg1* targeted with LoxP sites (hereafter termed "Arg1 KO" for simplicity). This system offers the significant advantage of efficient deletion of Arg1 in macrophages, the predominant cell type that expresses Arg1 outside the liver [11]. Our studies show that Arg1 in AAMs has no impact on the development of lung inflammation.

## Materials and Methods

### Mice, Genotyping, and Verification of Arg1 Deletion

All experiments performed at the National Institutes of Health used mice bred and housed under specific pathogen-free conditions in an American Association for the Accreditation of Laboratory Animal Care approved facility. The NIAID animal care and use committee approved all schistosome-related experimental procedures. Experiments performed at St. Jude Children's Research Hospital and USCF were approved by the corresponding Institutional Animal Care and Use Committees. The generation and genotyping of Arg1 KO mice has been described previously [17]. For schistosome egg and egg antigen experiments, C57BL/6 *Arg1* ^flox/flox^; Tie2-cre (Arg1 KO) mice were crossed with C57BL/6 *Nos2* ^−/−^ mice to generate *Arg1* ^flox/flox^ (wild-type) controls, *Arg1* ^flox/flox^; *Nos2* ^−/−^ (iNOS KO) mice, and *Arg1* ^flox/flox^; Tie2-cre; *NOS2* ^−/−^ (Arg1/iNOS KO) mice. For OVA, Aspergillus and NKT cell activation experiments, *Arg1* ^flox/flox^; Tie2-cre mice on a BALB/c background (n  = 6 generations for each allele) were used, or these mice were interbred with transgenic BALB/c mice expressing the chicken ovalbumin 323-339-specific DO11.10 TCR. The genotyping of Arg1-deficiency was initially verified using immunoblotting from cultured bone marrow macrophages. Bone marrow was collected at the experimental endpoint and cultured 7 days in media containing CSF-1. The resulting adherent macrophages were washed, stimulated overnight with 10 ng/mL each IL-4 and IL-10, and lysed in RIPA buffer. Arg1 was detected in macrophage lysates by immunoblotting with polyclonal anti-Arg1 antibodies as described [18].

### Schistosome Egg-induced Lung Inflammation

**Figure 1:**
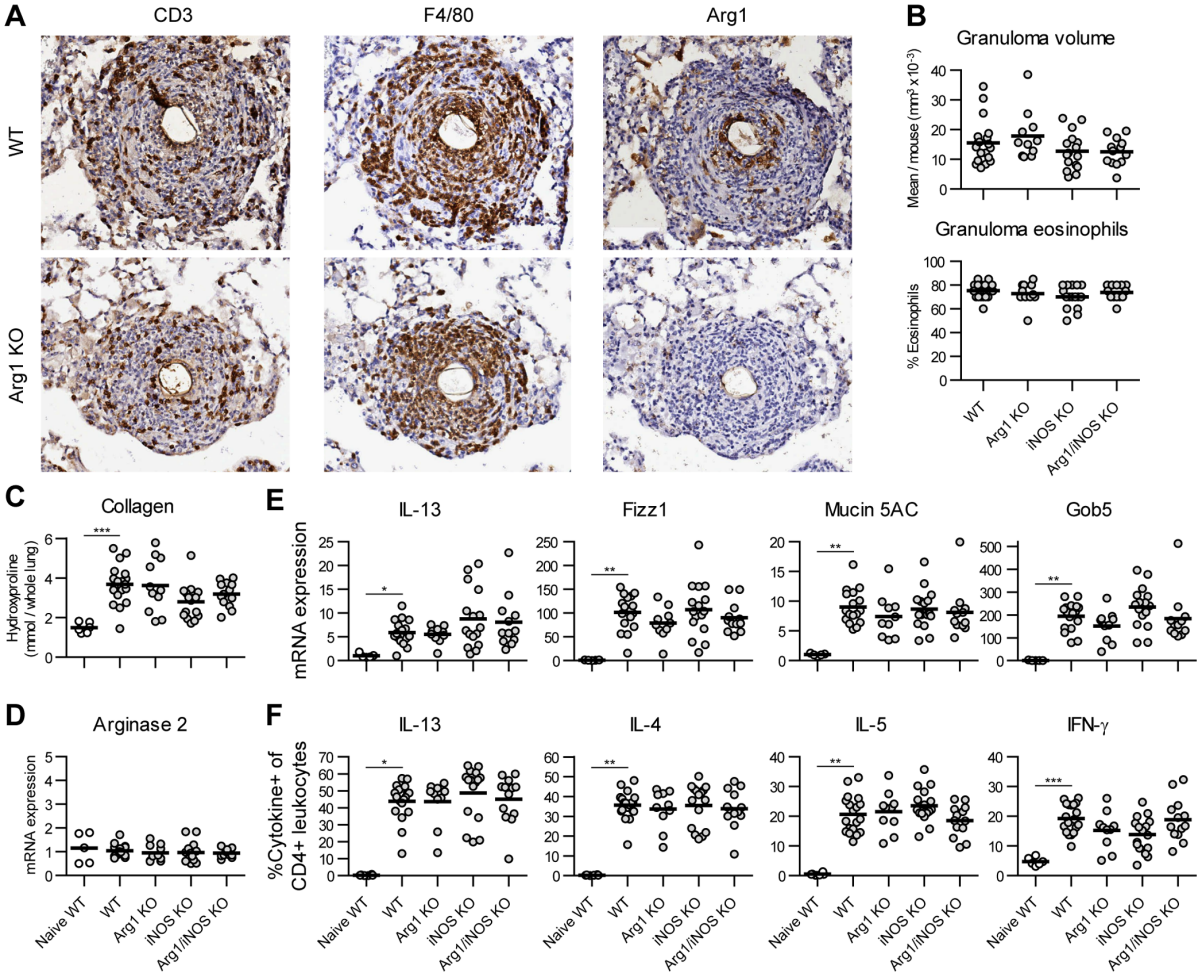
Arg1 expression by macrophages does not regulate acute schistosome egg-induced pulmonary granuloma formation and Th2 response. Mice deficient in Arg1, iNOS, or both enzymes were sensitized by i.p. injection of eggs, then challenged by a single i.v. injection of eggs to form pulmonary granulomas and analyzed at 2 weeks. A) T lymphocytes (CD3+), macrophages (F4/80+), and Arginase1+ cells were detected in fixed lung serial sections using immunohistochemistry. Images represent 8 mice of each genotype. B) The volume and eosinophil composition of 10–30 granulomas per mouse were scored in Geimsa-stained lung sections. C) Collagen deposition was compared by measuring L-hydroxyproline in matched lobes and calculating, by mass, total lung content. D) Expression of Arginase 2 or E) IL-13 mRNA and the Th2-responsive genes RELM-α, Mucin 5AC, and Gob5 were compared in lung tissue by quantitative PCR, normalized to the mean naïve WT level. F) To evaluate CD4 T cell responses, lung leukocytes were restimulated with PMA plus ionomycin and stained to detect IL-13, IL-4, IL-5, and IFN-γ by flow cytometry. No significant differences were observed between the challenged groups in B) to F). Results were combined from two independent experiments totaling 11 to 19 mice per group, plus 6 naïve WT controls. Individual mice and group means are shown.

**Figure 2:**
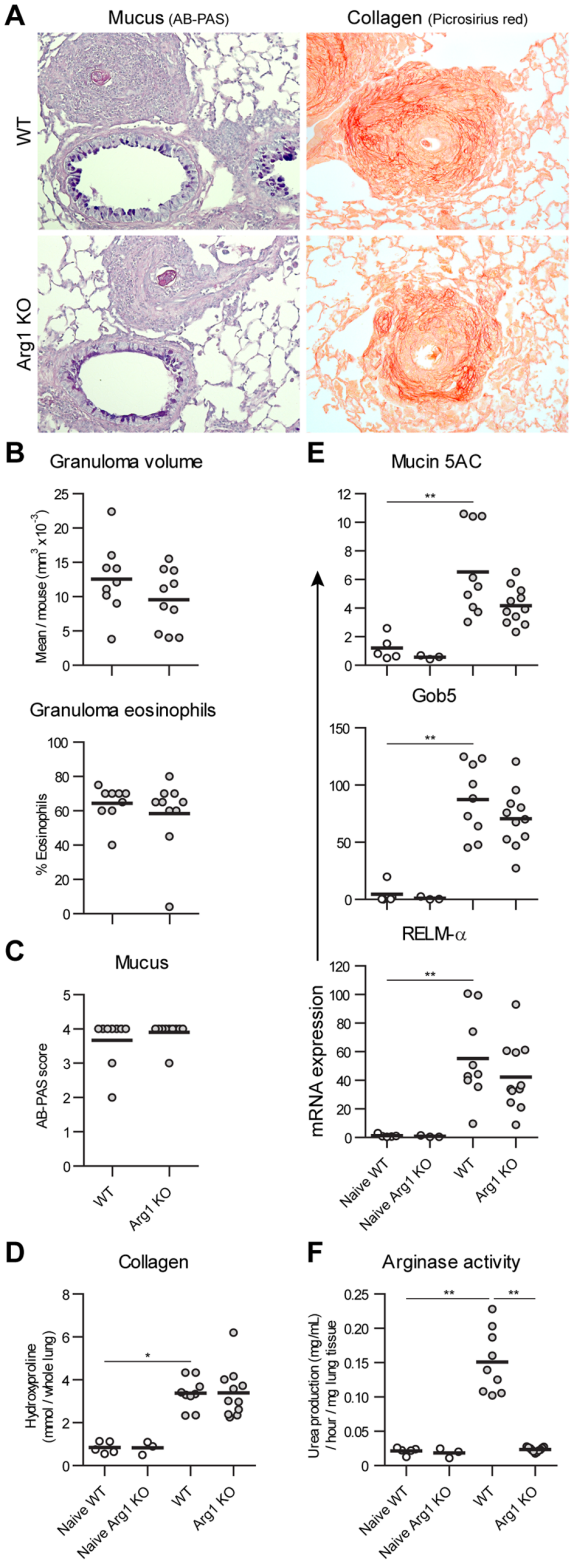
Arg1 expression by macrophages does not regulate chronic schistosome egg-induced pulmonary granuloma formation and Th2 response. Control and Arg1 KO mice were sensitized by i.p. injection of eggs, then challenged with 5 weekly doses of intravenous eggs to repeatedly form pulmonary granulomas and analyzed 2 weeks after the final challenge. A) The intensity and location of mucus (AB-PAS) and collagen (picrosirius red) were compared by histology in stained lung sections. Representative images of strong mucus production in airways adjacent to granulomas and dense collagen deposition around the perimeters of granulomas are shown. B) The volume and eosinophil composition of 6–29 granulomas per mouse were scored in Geimsa-stained lung sections. C) Cell-associated mucus around the airways was detected by AB-PAS staining and scored on a 0–5 point scale. D) Total lung collagen deposition was assessed by measuring L-hydroxyproline. E) Induction of mRNA transcription for the Th2-responsive genes RELM-α, Mucin 5AC, and Gob5 and was tested in lung tissue by quantitative PCR, normalized to mean expression in naïve WT mice. F) Arginase enzymatic activity was measured in lung tissue samples from naïve control and egg challenged mice. No significant differences were observed between challenged WT and Arg1 KO mice in A) to E). Results are from one experiment including 9 WT and 11 Arg1 KO challenged mice, plus 5 WT and 3 Arg1 KO naïve controls. Individual mice and group means are shown.

*Schistosoma mansoni* eggs were provided by the Biomedical Research Institute (Rockville, MD) [28]. For the acute lung granuloma model, mice were primed with intraperitoneal injection of 5,000 eggs in PBS, injected intravenously at week 2 with 5,000 live eggs in PBS to induce lung granulomas, and analyzed at week 4. For the repetitive lung granuloma model, primed mice received weekly i.v. injections of 1,000 live eggs at weeks 2 through 5, then 2,000 live eggs at week 6, and analyzed at week 8. To induce chronic airway inflammation, mice were injected i.p. with 5,000 eggs at weeks 0 and 2, then anesthetized with ketamine-xylazine and treated with 8 weekly intratracheal doses of 10 µg Soluble Egg Antigen, prepared as described [28], from weeks 4 to 11. SEA was delivered i.t. in 20 µL PBS with a gel loading pipette tip. Mice were analyzed 1 day after the final dose. Airway-infiltrating cells were recovered by bronchoalveolar lavage (BAL) with PBS plus 5 mM EDTA. Percentages of cell types were determined using cytospin preparations stained with Diff-Quick (Boehringer) by evaluating >200 cells/slide by light microscopy. For histological analyses, matched lung lobes were washed with PBS, inflated with Bouin’s fixative, and stained with Wright’s Giemsa. An experienced pathologist scored 6–30 granulomas per mouse (median 20, mean 20, standard deviation 6.5 for all data presented in Figures 1 and 2) to assess granuloma volume and cellular composition. For fibrosis, matched lung lobes were weighed and digested with HCl to measure the quantity of hydroxyproline [29], a characteristic component of collagens, or sections were stained with picrosirius red to evaluate collagen distribution. For mucus, fixed lung sections were stained with the Alcian Blue-Periodic Acid Schiff reaction to detect mucin polysaccharides associated with epithelial cells or the airway lumen, and scored by an experienced pathologist.

### Allergen-induced Lung Inflammation

For the ovalbumin model [30] mice were sensitized with i.p. injections of 50 µg OVA (Sigma-Aldrich) absorbed in 2 mg alum gel in 200 µL PBS on days 0, 7, and 14, then challenged intranasally with 100 µg OVA in 40 µL PBS on days 21, 22, and 23. For the Aspergillus model [31], mice received 100 µg of *Aspergillus fumigatus* (Hollister-Stier Laboratories) in 40 µl saline 3 times per week for 3 weeks. To measure airway reactivity [30] mice were anesthetized 24 h (OVA) or 48 h (Aspergillus) after the last challenge with ketamine (100 mg/kg of body weight), xylazine (10 mg/kg), and acepromazine (3 mg/kg) and the trachea cannulated with a 20 gauge tubing adaptor. Mice were attached to a ventilator and pulmonary mechanics analyzer (FlexiVent) and ventilated at 9 ml/kg tidal volume, 150 breaths/minute frequency, and 2 cmH_2_O positive end-expiratory pressure. Mice were paralyzed with pancuronium (0.1 mg/kg i.p.) and airway mechanics were measured continuously using the linear single compartment model while challenged with escalating doses of acetylcholine (0.03, 0.1, 0.3, 1 and 3 µg/g i.v.). BAL was performed via the tracheal cannula. Lungs were washed 3 times with 1 ml PBS, erythrocytes were lysed, and total BAL cells were counted with a hemocytometer. Percentages of cell types were determined using cytospin preparations stained with HEMA 3 (Fisher) by evaluating >300 cells/slide by light microscopy. Lavaged lungs were inflated with 10% buffered formalin to 25 cm H_2_O of pressure and fixed for 1 day. Multiple paraffin-embedded 5-µm sections of the entire mouse lung were prepared and stained with Hemotaxylin & Eosin (H&E) to evaluate the morphology and with Periodic acid-Schiff (PAS) to evaluate mucus production.

### NKT Cell-induced Inflammation

Mice received an intranasal dose of 100 ng PBS57 solubilized in DMSO plus OVA on day 0, followed by intranasal OVA alone on days 15, 16, 20 and 21, then sacrificed and analyzed 1 or 2 days after the final OVA dose. Each group of mice was divided and used for either BAL or histology.

### Immunohistochemistry

Antibody staining for Arg1 in paraffin embedded samples was optimized by semi-automated methods in the St. Jude Veterinary Pathology Core. Human liver obtained at autopsy was used as a positive control for human staining, while mouse livers generated from *Arg1* ^−/−^ mice were used as a negative control. Antibodies are described in figure legends.

### Arginase and iNOS Activity

Arginase activity was determined by incubating tissue or cell lysates with L-arginine and measuring urea production, as described [29]. Nitric Oxide Synthase activity was tested using the Griess reagent to measure nitrite concentrations in cell culture supernatants, as described [32].

### Quantitative PCR

Tissue samples were homogenized in Trizol using a Precellys24 (Bertin Technologies), immediately or after storage in RNAlater. RNA was extracted with chloroform, isolated with a MagMAX total RNA isolation kit, and reverse transcribed with Superscript II. Relative gene expression was measured by quantitative PCR amplification of cDNA using SYBR Green and an ABI Prism 7900HT Sequence Detection System (all from Life Technologies, Grand Island, NY) and normalized to RPLP2. All experiments used intron-spanning primers validated by dissociation curves, with these sequences: *RPLP2* (Fwd TACGTCGCCTCTTACCTGCT/Rev GACCTTGTTGAGCCGATCAT), *IL13* (CCTCTGACCTTAAGGAGCTTAT/CGTTGCACAGGGGAGTCT), *RELMa* (CCCTCCACTGTAACGAAGACTC/CACACCCAGTAGCAGTCATCC), *Muc5AC* (CAGGACTCTCTGAAATCGTACCA/AAGGCTCGTACCACAGGGA), *Gob5* (AGGAAAACCCCAAGCAGTG/GCACCGACGAACTTGATTTT), *Arg1* (GGAAAGCCAATGAAGAGCTG/GCTTCCAACTGCCAGACTGT), *Arg2* (TCCTCCACGGGCAAATTCC/GCTGGACCATATTCCACTCCTA), and *Nos2* (TGCCCCTTCAATGGTTGGTA/ACTGGAGGGACCAGCCAAAT).

### T Cell Restimulation and Flow Cytometry

Lung leukocytes were isolated from matched washed lobes crushed through a 100 µm cell strainer, or first minced and digested with 100 U/mL collagenase D (Sigma-Aldrich) while rocking for 60–90 at 37°, then underlaid with isotonic 36% Percoll (GE Healthcare), and centrifuged for 15′ at 350×g. Erythrocytes were eliminated with ACK lysing buffer (Quality Biological). BAL and lung leukocytes were counted using Trypan blue and a Cellometer Auto T4 (Nexcelom) or hemocytometer. To analyze cytokines, T cells were restimulated with 10 ng/mL PMA plus 1 µg/mL Ionomycin for 6 hours with 10 µg/mL Brefeldin A (Sigma-Aldrich) added for the final 2 hours of culture. Cells were washed, fixed in 2% paraformaldehyde, stored at −80°C in 10% DMSO, permeabilized with 0.5% saponin (Sigma-Aldrich) and stained with antibodies specific for CD4 (clone RM4-5, eBioscience), IL-4 (11B11, Biolegend), IL-13 (eBio13A, eBioscience), IL-5 (TRFK5, Life Technologies), and IFN-γ (XMG1.2, eBioscience). Alternatively, restimulated T cells were stained with Aqua live/dead (Invitrogen), washed, treated with Foxp3 fixation/permeabilization buffers (eBioscience) and stained for Foxp3 (FJK-16S, eBioscience), IL-10 (JES5-16E3, eBioscience), CD4 (GK1.5, Biolegend), CD45 (30F11, Biolegend), CD8α (53-6.7, Biolegend), and F4\80 (MF48-21, Invitrogen). Lung macrophages were analyzed using Aqua live/dead, Foxp3 buffers, and staining for CD45, F4\80 (BM8, Biolegend), CD11b (M1/70, Biolegend), Gr-1 (RB6-8C5, Biolegend), Siglec-F (E50-2440, BD Biosciences), Mannose Receptor (MR5D3, Biolegend), and RELM-α (226033, R & D Systems) labeled with Alexa Fluor 488 (Microscale Protein Labeling Kit, Life Technologies). Flow cytometry was performed using a FACSCanto II (BD Biosciences) and data were analyzed using FlowJo (Tree Star) using unstimulated and/or isotype controls of each sample to set gates.

### Total or OVA-specific IgE Assay

Sera were obtained from blood collected by cardiac puncture of antigen- or vehicle-treated mice. Total or OVA-specific IgE serum levels were measured by ELISA using plates coated with anti-mouse IgE (Pharmingen R35-72) or OVA. Diluted serum samples were added to each well, and bound IgE was detected with biotinylated anti-mouse IgE (Pharmingen, R35-118), streptavidin conjugated horseradish peroxidase (Pharmingen), and HRP substrate (TMB, BD Biosciences Pharmingen). Results are expressed as µg/ml IgE.

### Statistical Analyses

**Figure 3:**
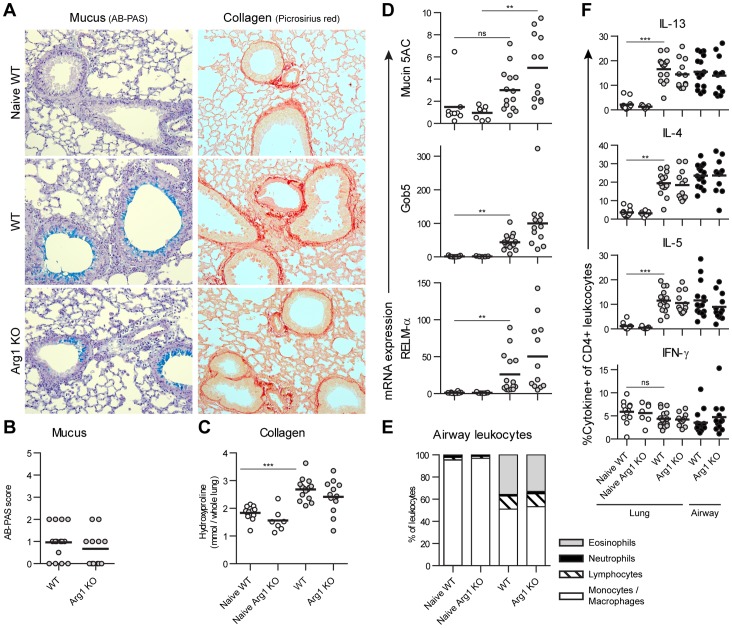
Arg1 expression by macrophages does not alter chronic SEA-induced airway inflammation. Arg1 KO mice were sensitized to schistosome eggs then treated with 8 weekly intra-tracheal challenges of soluble egg antigens to provoke chronic airway inflammation and analyzed 1 day after the final dose. A) The intensity and distribution of mucus (AB-PAS) and collagen (picrosirius red) were compared by histology in stained lung sections. Representative images of mucus-producing airway epithelium and underlying collagen deposition are shown. B) Mucus was visualized by AB-PAS staining and scored on a 0–5 point scale. C) Total lung collagen deposition was measured using L-hydroxyproline content. D) Induction of RELM-α, Mucin 5AC, and Gob5, and mRNA in lung tissue was compared by quantitative PCR, normalized to mean expression in naïve mice. E) Leukocytes from the airways were recovered by broncho-alveolar lavage and categorized by cytospin analysis. Cell numbers did not differ between WT and Arg1 KO mice (data not shown). F) Lung and airway leukocytes were restimulated with PMA plus ionomycin, stained, and analyzed by flow cytometry to compare cytokine production by CD4+ T lymphocytes. No significant differences were observed between challenged WT and Arg1 KO mice. Results were combined from two independent experiments totaling 12 WT and 7 Arg1 KO naïve mice, and 15 WT and 12 Arg1 KO challenged mice. Individual mice and group means are shown.

**Figure 4:**
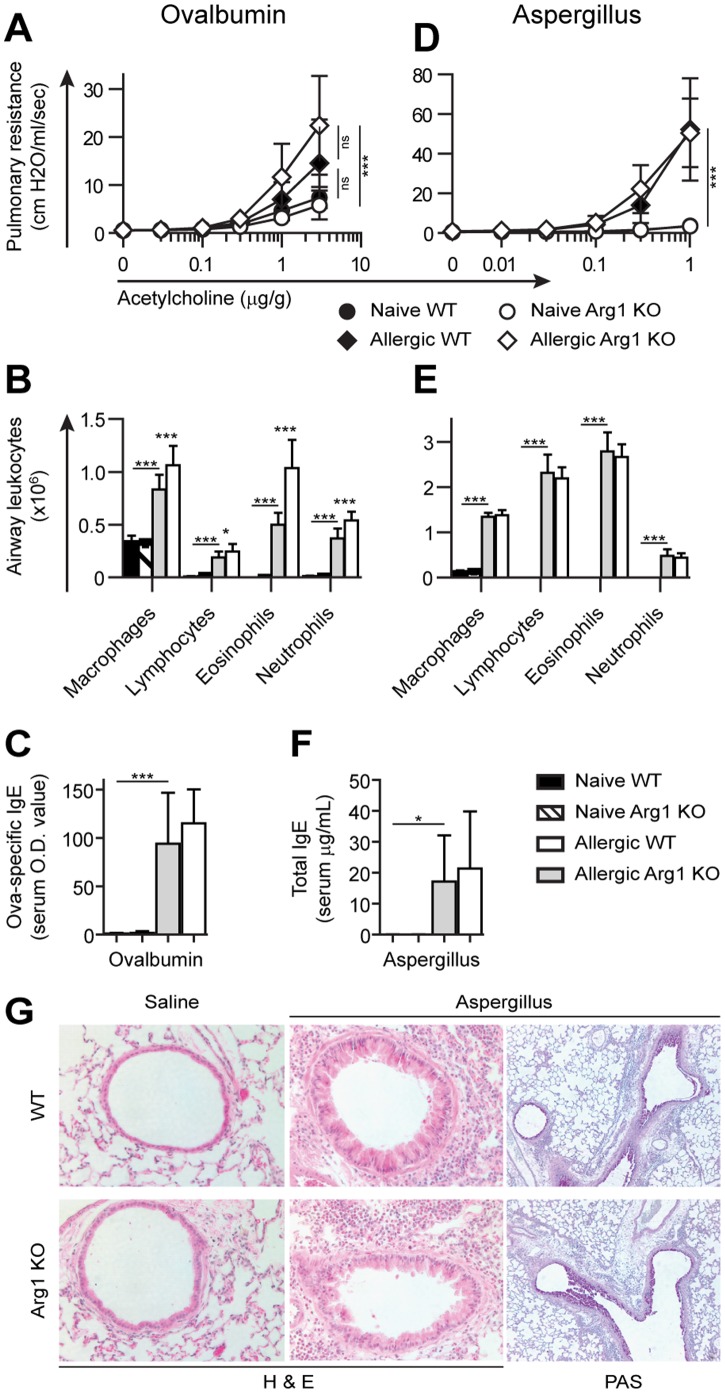
Arg1 expression by macrophages does not affect the outcomes of ovalbumin or Aspergillus-induced allergic airway and lung inflammation. A) WT and Arg1 KO mice were sensitized and challenged with intranasal Ova, then tested for airway hyper-reactivity. B) Airway-infiltrating leukocytes were recovered by lavage, counted, and categorized by cytospin analysis. C) Ova-specific serum IgE levels were compared by ELISA. D, E, F) Equivalent experiments compared the allergic response to Aspergillus. G) Aspergillus-induced lung inflammation was evaluated for eosinophil recruitment in eosin-stained sections and mucus production in PAS-stained sections. Representative histological images are shown. Except for the numbers of ovalbumin-induced inflammatory airway leukocytes, no significant differences were observed between challenged WT and Arg1 KO mice. Data represent 1 to 3 independent experiments including >8 mice/group, detailed in Table 1. Group means and standard deviations are shown.

Statistics were calculated with Prism (GraphPad Software, La Jolla, CA) applying 1-way ANOVA with Tukey’s multiple comparisons (Figure 1B), Kruskal-Wallis with Dunn’s post-test (Figures 1C–F, 2C–F, and 3), Mann Whitney (Figures 2B, S2, and S3), or 1-way ANOVA with Tukey’s multiple comparisons (Figure 4). Differences are noted as *(p≤0.05), **(p≤0.01), ***(p≤0.001), or ns (not significant).

**Table 1:** Experimental lung inflammation models performed in this study.

Assay	Route	# Exp	WT mice	#Arg1 KO	Site
**Acute ** ***S. mansoni*** ** eggs**	i.v.	2	19	11	NIAID
Eggs i.p. → wk 2 eggs i.v. → wk 4 end					
**Chronic eggs**	i.v.	1	9	11	NIAID
Eggs i.p. → wks 2, 3, 4, 5, & 6 eggs i.v. → wk 8 end					
**Chronic egg antigens**	i.t.	2	15	12	NIAID
Eggs i.p. → wk 2 eggs i.p. → wks 4, 5, 6, 7, 8, 9, 10, & 11 SEA i.t. → wk 11+ d 1 end					
**Ovalbumin**	i.n., aerosol	3	8–10 each	8–10 each	St. Jude
	i.n.	1	19	20	UCSF
Ova+alum i.p. d 0, 7, & 14 → Ova i.n. d 21, 22, 23 → d 24 end					
**Aspergillus**	i.n.	1	20	24	UCSF
Asp i.n. 3X wks 1, 2, & 3 → wk 3+ d 2 end					
**Ova+PBS57**	i.n.	3	8–10 each	8–10 each	St. Jude
	i.n.	1	12 DO11.10	4 DO11.10	St. Jude
Ova+PBS57 i.n. → Ova i.n. d 15, 16, 20, & 21 → d 22 or 23 end					

## Results

### The Subset of Arg1-expressing AAMs Cluster Around Th2 Inflammatory Sites

Previous studies discovered that Arg1 plays a protective role during *S. mansoni* infection by reducing the immune-mediated pathology induced when parasite eggs cross the intestinal barrier or become trapped in the liver, where Arg1+ macrophages surround the eggs in tightly clustered granulomas [32]–[34]. In chronic infections eggs may also become trapped in the lungs, forming Th2-dependent granulomas, if porto-pulmonary shunts develop in response to severe liver fibrosis. To investigate the role of Arg1+ AAMs in Th2-mediated lung pathology, we recreated, standardized, and synchronized this feature of schistosomiasis by intravenously injecting a single dose of live parasite eggs into egg-sensitized mice.

A Th2 immune response characterizes the lung granulomas surrounding schistosome eggs, with eosinophils, macrophages, and T lymphocytes forming a spherical inflammatory structure ringed by activated, collagen-producing fibroblasts (Figure 1A) [35]. While both CD3+ lymphocytes and F4/80+ macrophages were present throughout the granulomas and lung parenchyma, macrophages were predominantly localized in the granuloma centers. We screened anti-Arg1 antibodies for sensitivity and specificity in stained tissue sections (Figure S1) and evaluated Arginase 1 expression in lung granulomas by immunohistochemistry. Arg1+ cells were also found throughout the lung sections but concentrated centrally in the granulomas. However, as illustrated by serial sections, only a subset of the total F4/80+ macrophage population stained positive for Arg1 and these cells disproportionately clustered around the eggs in the granuloma cores. This distinction suggests heterogeneity in the type or activation status of macrophages even within the microenvironment of a granuloma dominated by Th2 cytokines.

### Arg1 Expression by AAMs does not Regulate Schistosome Egg-induced Lung Granuloma Formation or Th2 Responses

To test whether Arg1 plays a protective and immune-modulating role in the lung, as it does in the liver, we compared the acute granuloma formation, tissue remodeling and RNA profiles, and CD4+ T cell responses between WT and Arg1 KO mice in response to a single challenge with schistosome eggs. The nearly complete loss of Arg1 staining (Figure 1A) and arginase enzymatic activity (see below, Figure S2E) confirmed that this gene was efficiently targeted by Tie2-cre-mediated deletion in our mice and also, matching results from bone marrow chimera experiments [13], that increased Arg1 expression during lung inflammation derives from hematopoietic cells. Arg2 expression, which could perhaps compensate for the loss of Arg1 activity, did not change at the tissue mRNA level (Figure 1D).

In contrast to Arg1’s role in the liver and small intestine during schistosomiasis, we could not detect any distinctions between the lungs of WT and Arg1 KO mice acutely challenged with the same eggs and egg antigens (Figure 1). Two weeks after egg injection, granuloma size, eosinophil recruitment, and collagen deposition were unaltered by loss of Arg1 in AAMs in the lungs. Lung tissue mRNA levels of IL-13, and the Th2-inducible genes RELM-α, Mucin 5AC, and Gob5, all increased to a similar extent in WT and Arg1 KO mice, as did CD4+ T cell production of IL-13, IL-4, IL-5, and IFN-γ upon restimulation, Although Th2 cytokines predominated, we noticed elevated IFN-γ in T cells and NOS2 mRNA in the lung tissue (data not shown). The mutual antagonism between Arg1 and iNOS suggested that aberrant iNOS activity might mask the phenotype of Arg1 deletion. Arg1 deficiency did not alter the number of lung macrophages or otherwise impair their alternative activation, as assessed by RELM-α and Mannose Receptor expression, in egg-induced lung granulomas or egg antigen-induced airway inflammation (Figure S2). However, Arg1 KO lung leukocytes produced more nitrite when cultured alone or with LPS plus IFN-γ. To test a confounding role for iNOS in the absence of normal Arg1 expression, in the same experiments we also challenged mice lacking iNOS or both iNOS and macrophage-derived Arg1. However, deletion of iNOS or iNOS and Arg1 together revealed no phenotype (Figure 1).

We reasoned that a single, synchronous round of granuloma formation might proceed too quickly for Arg1-expressing AAMs to regulate the response. In Arg1 KO mice infected with *S. mansoni*, the early stages of liver inflammation and fibrosis proceed normally, but chronic egg-induced pathology is exaggerated because Arg1 negatively regulates persistent Th2 immunity [32]. We therefore treated egg-sensitized WT and Arg1 KO mice with 5 weekly i.v. injections of eggs, to repetitively drive pulmonary granuloma formation and prolong Th2 inflammation, and analyzed outcomes 2 weeks after the final dose. Arg1 deficiency caused no discernible changes in response to this chronic pulmonary challenge (Figure 2). Repeated egg challenge increased arginase enzymatic activity in lung tissue ∼7-fold over the naïve baseline, an increase entirely prevented in Arg1 KO mice. However, negating Arg1 induction did not alter the size and eosinophil composition of granulomas, collagen content, mucus production, or mRNA levels of RELM-α, Mucin 5AC, or Gob5, or in chronically inflamed lungs.

### Arg1 Expression by AAMs does not have an Essential Role in Regulating Chronic Airway Inflammation Induced by Schistosome Egg Antigens, or by Ovalbumin or Aspergillus

One consideration for these acute and chronic egg-induced granuloma experiments is that inflammation initiates at the endothelial surface because the eggs are trapped within the pulmonary vasculature. Although this process is a natural feature of chronic schistosomiasis, lung inflammation triggered by irritants, allergens, or infections generally originates at the airway epithelium. To determine whether Arg1 alters chronic airway inflammation, we sensitized WT and Arg1 KO mice with schistosome eggs, then challenged them with 8 weekly doses of intra-tracheal soluble egg antigens (SEA). Even after repetitively stimulating a Th2-mediated response through the airways we observed no substantial role for Arg1 in pulmonary immunopathology (Figure 3). Intra-tracheal SEA provoked Arg1-independent recruitment of eosinophils and lymphocytes into the airway, collagen deposition, mucus production, and transcription of the Th2-responsive genes for RELM-α, Mucin 5AC, and Gob5. Upon restimulation, lung and airway inflammatory CD4+ T cells equivalently produced IL-13, IL-4, IL-5, and IFN-γ in WT and Arg1 KO mice. In contrast, the same strain of Arg1-deficient mice exposed to the same egg antigens by infection in similar time frames develop an exaggerated Th2 response in the liver and intestine [32]. From these data, it appears Arg1 expression by AAMs causes organ-specific regulation of the immune response against schistosome eggs that reduces pathology in the liver and intestine but not the lungs.

*S. mansoni* egg antigens can downmodulate immune responses by activating Foxp3+ regulatory T cells and inducing IL-10, ameliorating the pathology of infection and reducing the response to other antigens in the lung [36]–[39]. Since this effect might compensate for Arg1 deficiency we assessed these populations of T cells in the lungs after challenge (Figure S3). Consistent with prior studies, both egg-induced lung granulomas and egg antigen-induced airway challenge recruited inflammatory Foxp3+ and IL-10+ CD4 T cells, but Arg1 deficiency did not alter these regulatory T cell populations.

To corroborate our findings, we performed similar allergic airway sensitization and challenge experiments with widely-employed antigens: ovalbumin (Ova) and Aspergillus. Ova-treated WT and Arg1 KO mice developed indistinguishable methacholine-induced airway hyperreactivity and elevated Ova-specific serum IgE levels (Figure 4). Although the composition of airway-infiltrating leukocytes did not differ, we recovered slightly more leukocytes from Ova challenged Arg1 KO than WT mice. Intra-nasal challenge with extracts from *Aspergillus fumigates* spores caused airway hyperreactivity and inflammation, IgE production, eosinophil recruitment, and mucus secretion, but control and Arg1 KO mice responded equivalently in each respect. In addition, we intensified the immune response to Ova by activating NKT or both NKT and Ova-specific CD4 T cells. We stimulated cytokine production from NKT cells using PBS57, a CD1d-binding glycolipid analog of α-galactosylceramide, while sensitizing mice to Ova [40]. Subsequent intra-nasal Ova treatment resulted in eosinophil-rich inflammation throughout the lungs yet without distinctions between WT and Arg1 KO mice (data not shown). We also bred control and Arg1 KO mice that expressed the DO11.10 transgenic T cell receptor, so that approximately half their CD4 T cells would react to Ova. Treatment with PBS57 plus Ova provoked extensive pulmonary inflammation in these mice but, again, no apparent Arg1-dependent distinctions (data not shown). Thus, besides minor differences in the number of airway leukocytes in one experimental protocol, we find that Arg1 expression by myeloid cells does not regulate pulmonary immunopathology in six different models of Th2-dependent lung inflammation.

## Discussion

### Macrophage-derived Arg1 is not a Key Factor in Murine Th2 Lung Inflammation and Asthma Models

Arginase inhibition is an appealing and novel strategy to treat asthma. Arginase activity in the lungs has been associated with pathology, and existing arginase inhibitors seem relatively non-toxic and could potentially be administered via aerosol [20], [22], [41], [42]. Indeed, inhibitor studies in guinea pigs and siRNA-mediated interference experiments in mice argue that inhibiting arginases reduces Th2-induced lung pathophysiology [23], [24].

How might Arg1 regulate asthma and lung inflammation? Current evidence presented a minimum of four hypothetical and possibly linked scenarios. (i) Arg1 reduces NO production by competing with nitric oxide (NO) synthases for arginine, their common substrate, [43], [44] as well as affecting iNOS translation [45]. Arg1 has a lower affinity for arginine than iNOS, but competes effectively at a biochemical level because of its faster catalytic rate [10]. NO plays an important role in airway physiology, lung inflammation, and host defense, and Arg1 can inhibit NO production both in vivo and in vitro [17], [43], [44]. However, macrophages generate NO after upregulating iNOS in response to TLR and interferon signaling [46]. Since a Th2 response drives asthmatic lung inflammation it seems unlikely that AAMs in the lung would express substantial iNOS, although this possibility has not been ruled out. Arg1 could instead indirectly limit arginine availability to other NOS isoforms expressed by non-hematopoietic cells in the lung.

(ii) Arg1 can supply metabolites for synthesizing collagen and polyamines, thereby potentially affecting tissue remodeling. The arginase reaction yields ornithine, the source of substrate for proline, which is essential to produce collagens. Ornithine is also the sole source of substrate for polyamine synthesis [10]. By regulating these metabolic pathways, Arg1 could cause important changes in the function, inflammation, remodeling, or fibrosis of the lung parenchyma and airways. In support of this idea, IL-4/13 stimulates proline production by bone marrow-derived macrophages and the availability of ornithine to these AAMs limits proline output [47].

(iii) Arg1 controls overall arginine bioavailability in the lung [22]. The amount of arginase activity in vivo depends on elevated transcription of *Arg1,* and possibly *Arg2*, coupled to the rate of active transport of arginine into cells [9], [10]. Expression of one arginine transporter, CAT2, is also increased by Th2 cytokines [12], and CAT2-deficient mice spontaneously develop lung inflammation [48]. Therefore, it is plausible that the rate of arginine hydrolysis could increase by orders of magnitude in a local environment as Th2 inflammatory signals coordinately boost enzyme synthesis, substrate import, and other limiting factors. Reduced arginine concentrations could limit substrate for NO synthases in non-myeloid cells as well as alter the metabolic balance in the lung.

(iv) By depleting arginine Arg1 blocks T cell proliferation. The importance of this mechanism was demonstrated in chronic schistosomiasis, an infection causing substantial organ damage but little mortality. Arg1+ AAMs form granulomas in the liver and intestine around the parasite eggs and restrain the immune response against these eggs. If Arg1 is deleted in AAMs, granuloma size increases, egg-induced pathology becomes lethal, and, in vitro, AAMs impair T cell proliferation in an Arg1-dependent mechanism that can be overcome by supplying extra L-arginine [32], [33].

By contrast, here we demonstrate that Arg1 expression by macrophages plays neither a protective nor pathogenic role in a diverse range of Th2-mediated lung inflammatory insults. Our results, taking a genetic approach to generate macrophages devoid of *Arg1*, agree with a recently published study where lethally-irradiated mice reconstituted with *Arg1*-deficient bone marrow exhibited normal airway function and lung inflammation [13]. Since irradiation has a profound effect of normal lung function [49], it was possible that additional Arg1-independent effects may have obscured the outcomes of Th2-driven inflammation in that setting. Thus, our genetic-based data substantiate the results of Neise et al [13]. Although arginase inhibitors affect both Arg1 and Arg2 [9], [41], [42], Arg1 expression by hematopoietic cells accounts for allergen-induced increased in arginase activity in the lungs, and eliminating Arg2 had no effect [13]. Furthermore, we found that eliminating iNOS in combination with Arg1 also had little effect, suggesting that the Arg1-iNOS nexus in macrophages is dispensable for all obvious phenotypes of Th2-driven lung inflammation. Accordingly, we argue that drugs targeting arginases in the lung should be evaluated with considerable caution.

### Inhibitory Effects of Macrophage-derived Arg1 in Th2 Responses are Organ-specific

Our results with lung inflammation models, including schistosome egg and egg antigen-induced inflammation, are strikingly juxtaposed to Arg1’s essential role in enabling macrophages to restrain the hepatic and intestinal Th2 response in schistosome-infected mice [32], [33]. In all three tissues Arg1-positive AAMs surround the egg and interact with T cells in a granuloma microenvironment. In the liver, however, Arg1-positive macrophages suppress excessive Th2 responses (and perhaps other types of T cell responses [33]), decreasing granuloma size, fibrosis, and hepatomegaly, and even preventing death. Since Arg1-expressing macrophages restrict T cell proliferation in vitro and in schistosome-infected mice [32], [33], we hypothesized that arginine depletion by AAMs might function as a feedback mechanism for regulating immunity. Th2 immune responses recruit and alternatively activate macrophages, increasing arginase activity, decreasing arginine availability, and therefore enabling Arg1 to control T cell proliferation and function within a localized tissue environment [50]. In the lungs, by contrast, we observed no differences in T cell phenotype in Arg1 KO mice even though the immune response to eggs in the lungs is ostensibly identical to that in the liver.

We hypothesize that organ differences in Arg1 function depend on the rate and/or magnitude of circulating arginine. Liver tissue has high cellular density, high baseline arginase activity, and, by late stage schistosomiasis, severely impaired perfusion. In contrast, even chronically inflamed lung tissue has (compared to liver) low cellular density, low arginase activity, and largely intact perfusion, which to us seems a plausible explanation for why T cells in the lung do not experience arginine deprivation that triggers cell cycle arrest. While simplistic, this concept may predict the environments where arginine deprivation impacts immunity and pathology: in the liver, kidney, and small intestine where parenchymal arginase activity is high, in solid tumors infiltrated with myeloid-derived suppressor cells, and in fibrotic tissues (Figure S4). Analogous predictions may apply to depletion of other critical metabolites, such as tryptophan and cysteine. At this stage, however, the technical requirements for measuring the quantity of free arginine available for uptake by cells within tissue microenvironments prevents direct testing of this hypothesis.

### Why is Arg1 Induced by Macrophages in the Th2-inflammed Lung if it is not Required?

It is important to consider that Arg1 is only one of the suite of genes induced in AAM, and AAM are only one element of the immune response coordinated by Th2 cytokines [11]. IL-4 and IL-13 induce a predictable pattern of gene expression in macrophages, regardless of the associated infection or disease [3]. Increased Arg1 is considered a canonical marker for mouse AAMs and is found in virtually every scenario where Th2 cytokines stimulate macrophages [11]. Recent data prove that Arg1 can also be expressed in macrophages in many contexts distinct from a Th2 response [17], [18]. Thus, while mouse AAM express Arg1, not all Arg1 positive macrophages are alternatively activated.

Since Th2-driven AAM gene expression response is apparently activated regardless of the infection or insult, some of these genes may not play important roles in every situation. Mice with a selective deletion of the IL-4Rα in macrophages mount a normal CD4+ T cell Th2 response but, since their macrophages cannot bind IL-4 or IL-13, they specifically lack AAMs. Infection of these mice demonstrated that AAM critically affect immune reactions to schistosomes, but are dispensable for immunity to the parasitic nematode *Nippostronglyus brasiliensis*, which is also controlled by a Th2 response [51]. Such differences may be explained if it is favorable to elicit the entire AAM gene expression profile even though only a subset (or one) gene product is necessary for effective macrophage-mediated immunity. Thus, we argue that increased expression of Arg1 in macrophages during asthma and lung inflammation is a consequence of a pre-set activation program necessary to combat a wide variety of parasites (especially worms) in a tissue-specific way. In some cases, Arg1 is obligatory for immunity or to protect the host but, in other situations, other Th2-induced gene products may be needed.

Furthermore, Th2-induced genes that distinguish AAMs from macrophages in different activation states can also be upregulated by other cell types [3]. Redundant or compensatory expression of key molecules by multiple cell types could explain why allergic airway disease was recently and, to us, surprisingly, found to be unaffected in LysM-Cre IL-4Ra^Flox^ mice in which macrophages cannot be activated by IL-4 or IL-13 [27]. For Arg1, we suggest that expression by fibroblasts might prove important for lung disease, since cell intrinsic arginase activity could promote fibrosis via proline to collagen and polyamine synthesis. This hypothesis may explain why deleting Arg1 in macrophages does not alter Th2-mediated lung inflammation or pathology while arginase inhibitors or siRNA nonetheless ameliorate some features of asthma.

Our results demonstrate that a major obstacle to understanding the roles of AAMs will be distinguishing correlation from causation. Indeed, detecting associations between Arg1, or any other IL-4/13-stimulated gene in macrophages, and host defense or immunopathology merely demonstrates a Th2 immune response. Only by testing putative macrophage effector function genes one at a time against a variety of challenges will we gain a full understanding of the overall complexity and robustness of the AAM response.

However, the heterogeneity within macrophages populations offers an alternative experimental approach. In this study, we anticipated that nearly all macrophages inside a Th2-derived granuloma would express Arg1. Instead, even within this inflammatory microenvironment, most macrophages appear not to express Arg1 unless located at the core, near the schistosome egg. Our data suggest that, even over very short distances, macrophages have differential access to IL-4, IL-13, and other activation signals. Careful dissection of the combinations of macrophage-activating signals offers a complementary and potentially more productive strategy than iterating the approach we took with Arg1 with every interesting gene STAT6 induces in macrophages.

## Supporting Information

Figure S1 **Evaluation of anti-Arg1 antibodies.** The sensitivity and specificity of commercially available mono- and polyclonal anti-Arg1 antibodies were tested by immunohistochemical staining of fixed human and mouse liver sections. We selected the sc-20150 anti-mouse Arg1 antibody for use in experiments after comparing the staining intensities of hepatocytes (positive control), liver endothelium (no Arg1 expression), and entirely Arg1-deficient mouse liver (negative control).
(TIF)

Figure S2 **Effects of Arg1 deficiency on macrophage phenotypes.** Control and Arg1 KO mice were sensitized by i.p. injection of eggs, then challenged with either intravenous eggs to induce lung granulomas or intra-tracheal SEA to cause airway inflammation. Leukocytes were isolated from perfused and digested lungs, and macrophages were analyzed by flow cytometry. Macrophages were identified by gating on live CD45+ Siglec F(neg/low) Gr1(neg/low) F4\80+ CD11b+ events and stained for RELM-α and mannose receptor expression, as markers of alternative activation. Representative samples of A) d8 lung granuloma and B) d7 airway SEA mice challenged on d0 and d6 are shown. C) Percentages and numbers of macrophages, and D) expression levels of RELM-α and mannose receptor were calculated for group means and individual mice. Alternatively, lung leukocytes were cultured overnight with no added stimulation, 20 ng/mL IL-4, or 2 mg/mL LPS plus 20 ng/mL IFN-γ. E) Arginase activity was measured in cell lysates by urea production and F) iNOS activity was measured by nitrite production in culture supernatants, normalized to the input number of macrophages and shown as group means and standard deviations.
(PDF)

Figure S3 **Effects of Arg1 deficiency on regulatory CD4+ T lymphocytes.** Control and Arg1 KO mice were sensitized and challenged as described in Figure S2. Lung leukocytes were stimulated with PMA, Ionomycin, and Brefeldin A, or cultured with Brefeldin A alone, and analyzed by flow cytometry. CD4+ T cells were identified by gating on live CD45+ CD4+ CD8α(neg) F4\80(neg) events and stained for Foxp3 and IL-10 expression. Representative samples of A) d8 lung granuloma and B) d7 airway SEA mice challenged on d0 and d6 are shown. C) Numbers of CD4 T lymphocytes and percentages of Foxp3+ cells, and D) IL-10 expression by Foxp3(neg) and Foxp3+ CD4+ T lymphocytes were calculated for group means and individual mice.
(PDF)

Figure S4 **Arginase activity in different tissues.** Samples of liver, kidney, small intestine (subdivided into duodenum, jejunum, and ileum), colon, lung, spleen, and mesenteric lymph nodes were taken from 4 perfused naive mice. Tissue arginase activity was measured by urea production.
(TIF)
