# Supplementary figures and images for: Role of Arginase 1 from Myeloid Cells in Th2-Dominated Lung Inflammation
Source: PLoS One. 2013 Apr 24;8(4):e61961. doi: 10.1371/journal.pone.0061961 (PMC3634833; doi:10.1371/journal.pone.0061961)

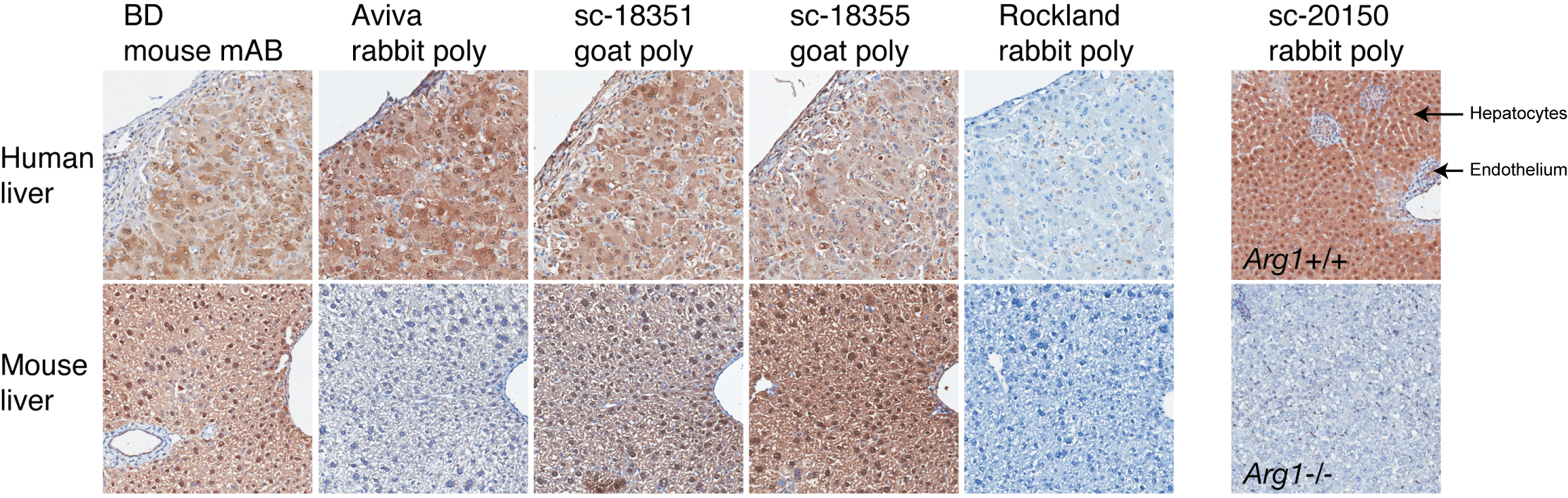

Supplement: Figure S1 — Evaluation of anti-Arg1 antibodies. The sensitivity and specificity of commercially available mono- and polyclonal anti-Arg1 antibodies were tested by immunohistochemical staining of fixed human and mouse liver sections. We selected the sc-20150 anti-mouse Arg1 antibody for use in experiments after comparing the staining intensities of hepatocytes (positive control), liver endothelium (no Arg1 expression), and entirely Arg1-deficient mouse liver (negative control). (TIF) [file pone.0061961.s001.tif]

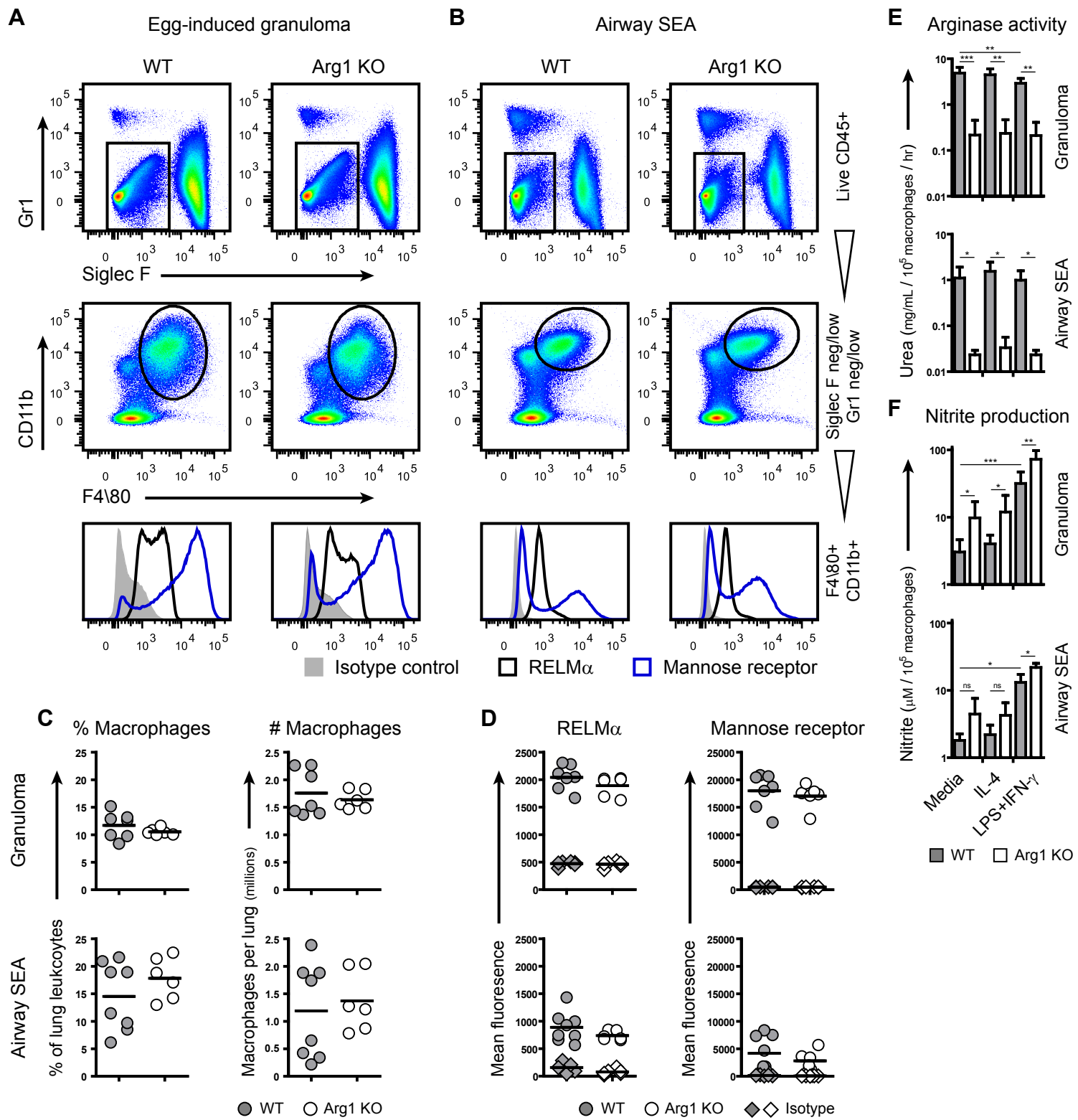

Supplement: Figure S2 — Effects of Arg1 deficiency on macrophage phenotypes. Control and Arg1 KO mice were sensitized by i.p. injection of eggs, then challenged with either intravenous eggs to induce lung granulomas or intra-tracheal SEA to cause airway inflammation. Leukocytes were isolated from perfused and digested lungs, and macrophages were analyzed by flow cytometry. Macrophages were identified by gating on live CD45+ Siglec F(neg/low) Gr1(neg/low) F4\80+ CD11b+ events and stained for RELM-α and mannose receptor expression, as markers of alternative activation. Representative samples of A) d8 lung granuloma and B) d7 airway SEA mice challenged on d0 and d6 are shown. C) Percentages and numbers of macrophages, and D) expression levels of RELM-α and mannose receptor were calculated for group means and individual mice. Alternatively, lung leukocytes were cultured overnight with no added stimulation, 20 ng/mL IL-4, or 2 mg/mL LPS plus 20 ng/mL IFN-γ. E) Arginase activity was measured in cell lysates by urea production and F) iNOS activity was measured by nitrite production in culture supernatants, normalized to the input number of macrophages and shown as group means and standard deviations. (PDF) [file pone.0061961.s002.pdf]

**A**

Egg-induced granuloma

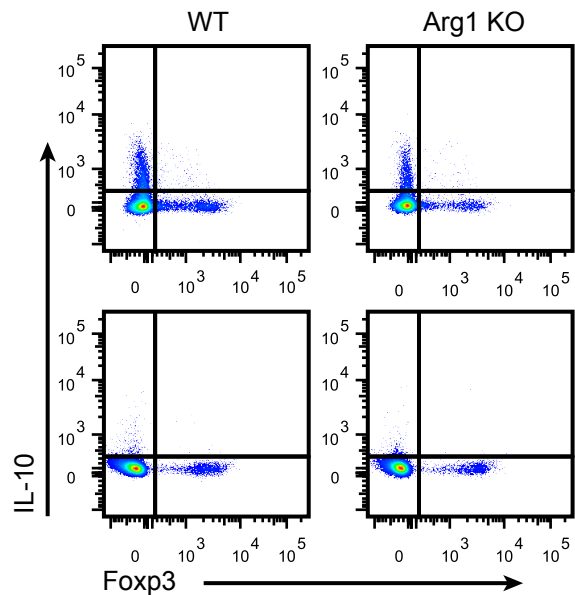**B**

Airway SEA

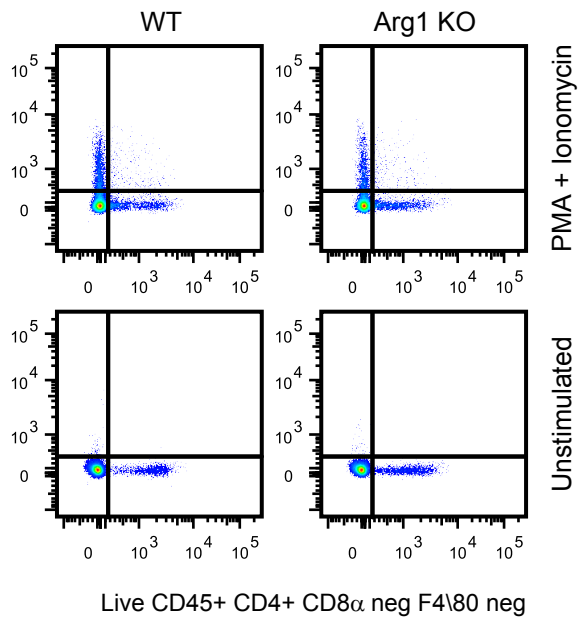**C**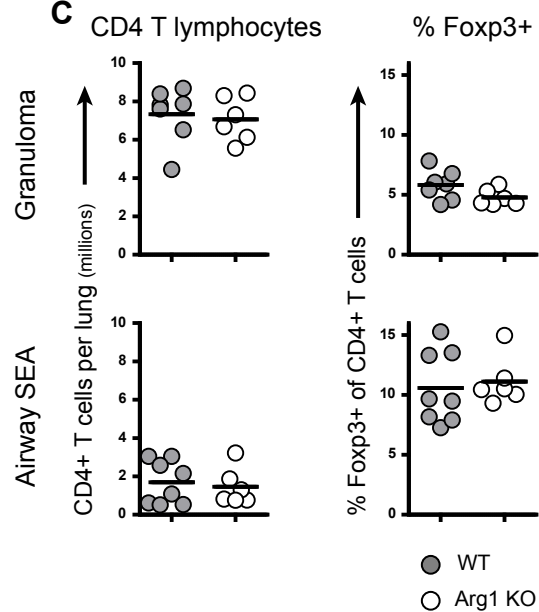**D**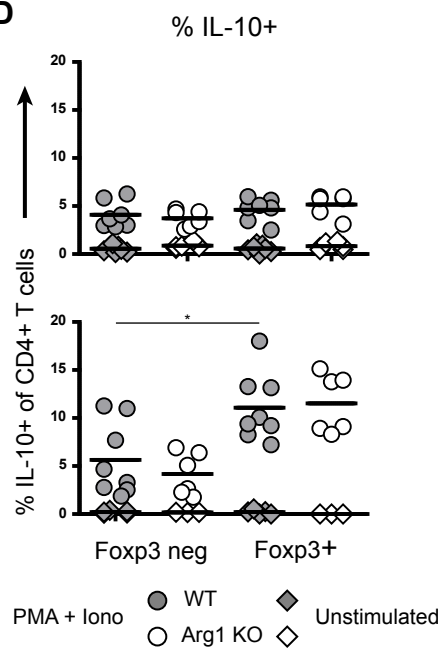

Supplement: Figure S3 — Effects of Arg1 deficiency on regulatory CD4+ T lymphocytes. Control and Arg1 KO mice were sensitized and challenged as described in Figure S2. Lung leukocytes were stimulated with PMA, Ionomycin, and Brefeldin A, or cultured with Brefeldin A alone, and analyzed by flow cytometry. CD4+ T cells were identified by gating on live CD45+ CD4+ CD8α(neg) F4\80(neg) events and stained for Foxp3 and IL-10 expression. Representative samples of A) d8 lung granuloma and B) d7 airway SEA mice challenged on d0 and d6 are shown. C) Numbers of CD4 T lymphocytes and percentages of Foxp3+ cells, and D) IL-10 expression by Foxp3(neg) and Foxp3+ CD4+ T lymphocytes were calculated for group means and individual mice. (PDF) [file pone.0061961.s003.pdf]

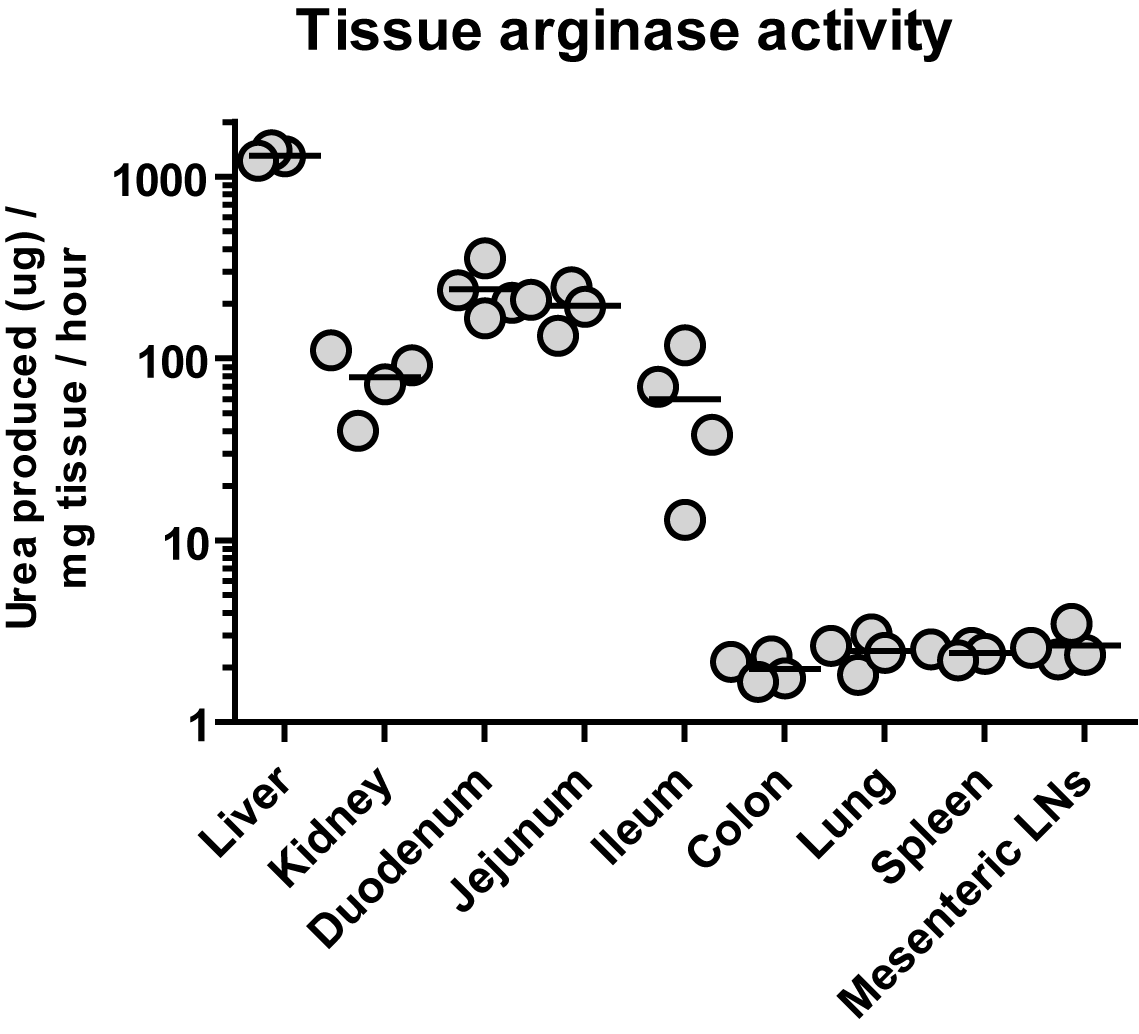

Supplement: Figure S4 — Arginase activity in different tissues. Samples of liver, kidney, small intestine (subdivided into duodenum, jejunum, and ileum), colon, lung, spleen, and mesenteric lymph nodes were taken from 4 perfused naive mice. Tissue arginase activity was measured by urea production. (TIF) [file pone.0061961.s004.tif]
